# Spidroin-Based Biomaterials in Tissue Engineering: General Approaches and Potential Stem Cell Therapies

**DOI:** 10.1155/2021/7141550

**Published:** 2021-12-20

**Authors:** Qi Zhang, Min Li, Wenbo Hu, Xin Wang, Jinlian Hu

**Affiliations:** ^1^Department of Biomedical Engineering, City University of Hong Kong, Kowloon, Hong Kong; ^2^Biological Science Research Center, Southwest University, Chongqing 400716, China

## Abstract

Spider silks are increasingly gaining interest for potential use as biomaterials in tissue engineering and biomedical applications. Owing to their facile and versatile processability in native and regenerated forms, they can be easily tuned via chemical synthesis or recombinant technologies to address specific issues required for applications. In the past few decades, native spider silk and recombinant silk materials have been explored for a wide range of applications due to their superior strength, toughness, and elasticity as well as biocompatibility, biodegradation, and nonimmunogenicity. Herein, we present an overview of the recent advances in spider silk protein that fabricate biomaterials for tissue engineering and regenerative medicine. Beginning with a brief description of biological and mechanical properties of spidroin-based materials and the cellular regulatory mechanism, this review summarizes various types of spidroin-based biomaterials from genetically engineered spider silks and their prospects for specific biomedical applications (e.g., lung tissue engineering, vascularization, bone and cartilage regeneration, and peripheral nerve repair), and finally, we prospected the development direction and manufacturing technology of building more refined and customized spidroin-based protein scaffolds.

## 1. Introduction

**Figure 1:**
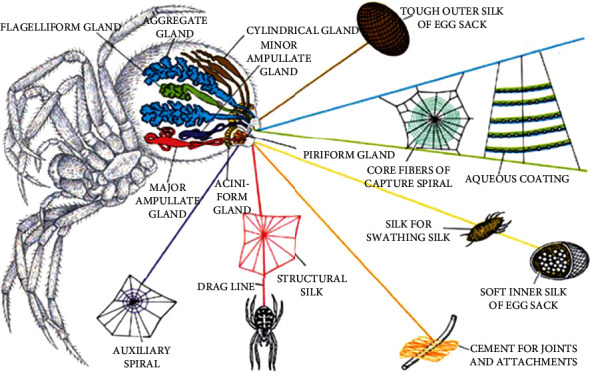
Seven types of spider silk glands and spider silks from spider *Araneus diadematus*: dragline/major ampullate silk (red), minor ampullate silk (purple), flagelliform silk (green), tubuliform silk (brown), aciniform silk (yellow), aggregate silk (blue), and piriform silk (orange). Reproduced from F. Vollrath, 2000, Copyright © 2000, Elsevier Science B.V. All rights reserved.

Spider silk has attracted much attention for thousands of years due to its resilience, toughness, and biocompatibility. Since ancient Greeks, natural spider silks have been well documented in medical applications as bandages owing to their outstanding hemostatic performance [1, 2]. The antibacterial phenomena and hypoallergenic properties have also been found from natural spider silks [3], which indicate spider silk material's great potential in biomaterial and tissue engineering areas. In nature, spiders produce various types of silk fibers from separate glands for multiple purposes of creating shields for protection from predators, encasing their eggs, and weaving webs. As shown in Figure 1, up to seven types of silk-producing glands extrude proteins through spinnerets to produce spider silks, e.g., dragline/major ampullate silk, minor ampullate silk, flagelliform silk, tubuliform silk, aciniform silk, aggregate silk, and piriform silk. Each type of silk fiber presents unique mechanical properties, internal structure, and composition, all determined by their functions [4, 5]. Among these kinds of spider silks, the major ampullate silk (also called dragline silk) has been extensively investigated due to its excellent comprehensive mechanical properties [6, 7]. Compared with natural fibers and synthetic materials, spider silks exhibit a well-balanced combination of stiffness and elasticity, which perfectly satisfies scaffold biomaterial's mechanical and biological requirements for tissue engineering. However, the natural sources of spider silks and processing methods are limited. Therefore, technological strategies in genetic engineering have long been used to integrate specific genes and recombinantly engineered natural spider silk proteins.

Spider silk is a protein biopolymer that varies in physicochemical and biological properties due to a variation in structure and composition. Based on this processability, researchers manage to use natural or recombinant spider silk proteins as raw material to manufacture tailor-made scaffolds with controllable structures and outstanding properties for diverse tissues or applications.

As an essential part of tissue engineering, scaffolds are usually used for functional reconstruction of damaged organs and defective tissues, including promoting cell adhesion and proliferation, extracellular matrix regeneration, and recovery of vessels and nerves around newly generated tissues, which requires that scaffold materials should be processible to be manufactured into different forms and structures to provide sufficient space for substance and information interexchange between tissue and cells. In addition, scaffolds should also have good biocompatibility to reduce inflammation and cytotoxicity. As the center of tissue regeneration and carrier of stem cells and cell signals, the corresponding mechanical strength of scaffold materials should be also guaranteed to maintain the complex structure of the scaffold during tissue regeneration [8, 9]. For many biomedical applications, spidroin has been modified to form specific structures, including thin films, fibers, hydrogels, microspheres, and sponges. And the further understanding of the internal characteristics of spider silk protein structure-function will also help to design new polymer materials inspired by nature.

**Figure 2:**
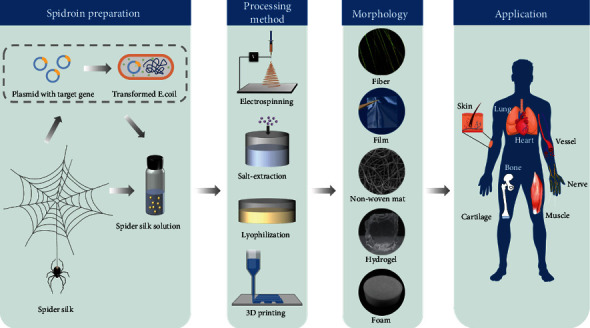
Schematic diagram of scaffold preparation using recombinant spidroins manufactured by transformed *E. coli* and their applications in the tissue engineering area. Reproduced from S. Salehi et al., 2020, under the Creative Commons Attribution License.

In this review, we will discuss spidroin-based scaffolds with different dimensions and their applications in tissue engineering and regenerative medicine as shown in Figure 2, to demonstrate the great potential of spidroin-based material in the biomedical engineering field. Because of the complexity of lung tissue regeneration, there is a lack of research on the application of spidroin-based materials to lung tissue repair. What is exciting is that we still see the great possibility of using spidroin-based biomaterials for lung tissue engineering from many researches on spidroin-based scaffolds, and they are also summarized in this review. Meanwhile, we also make an outlook about the development of the next-generation multifunctional spidroin-based scaffold through the manufacturing process, genetic engineering, and molecular biology.

## 2. Spider Silk as a Matrix for Tissue Engineering

### 2.1. Extracellular Matrix (ECM) and Requirement for Scaffold Design

The extracellular matrix (ECM) is the tissue-specific three-dimensional network architecture on or between cells. It is mainly composed of cell secretory products in each tissue and organ, including collagen, fibronectin, laminin, glycosaminoglycan, and growth factor. Each component has its own role and function. As the most abundant protein in ECM, collagen provides the connection between ECM and actin cytoskeleton to promote the exchange of nutrients and information. In general, ECM not only offers the necessary structural strength required for common tissue activities but also adjusts the cell living, apoptosis, proliferation, differentiation, shaping, and migration through growth factors and cytokines [10–13].

For scaffold design in tissue engineering, many factors should be carefully considered to mimic the ECM function and create a favorable living environment for tissue and organ regeneration [11]. Firstly, biocompatibility and biosafety are particularly important to scaffold material. There should be no cytotoxicity and immunoreaction when scaffolds are implanted into the human body [14, 15]. Secondly, the appropriate mechanical properties of scaffolds also need to be emphasized. During treatment, most scaffolds need to temporarily bear tension or pressure to remodel and induce the differentiation of stem cells, so their mechanical properties are preferred to be similar with surrounding tissues [16]. Thirdly, the processability of biomaterial is also necessary, which allows biomaterials to be precisely designed and processed into various effective structures. In order to simulate the ECM function mentioned above, porous or fibrous architecture is more suitable for intercellular communication and nutrient transport. On the one hand, this scaffold architecture has relatively high specific surface area, which offers more connecting sites for fibronectin proteins to adhere to stem cells. On the other hand, a porous structure scaffold with appropriate porosity provides space for neovascularization and the nervous system and reconstructs metabolic and information channels between new tissues and their surrounding [11, 17, 18]. Finally, the degradation rate of the scaffold should match the rate of secretion and generation of new ECM and tissue, and this processing rate can be adjusted through modifying scaffold composition, structure, etc. Meanwhile, the degradation products must also be noncytotoxic to ensure that there will be no secondary damage due to degradation in the process of tissue regeneration. With the development on the research about tissue engineering and interaction between biomaterials and tissues, higher and higher requirements are put forward for scaffold design, which is both a challenge and an opportunity to provide better scaffolds for tissue engineering [19, 20].

### 2.2. Interactions between Spider Fibroin and Cells

Over the past years, researchers have generated extensive evidence that biochemical cell-matrix interactions can improve cell survival and function. Many approaches, including chemical modification of the primary structure of spidroin, gene modification with cell-binding motifs, and the change of the material surface topographies, have been adapted to improve cell behaviors (e.g., cell attachment, proliferation, and differentiation) to scaffolds made of spider silk proteins [21–24].

Chemical coupling techniques rely on certain chemicals that are not always biocompatible with cells, and the efficiency of this nonspecific adhesion on *in vitro* culture is relatively low for some types of cells [25]. To further improve the interactions between cells and biomaterials, the cell-binding peptide motif, such as RGD (Arg-Gly-Asp), has been extensively incorporated in materials. Widhe et al. demonstrated the viability of fibroblast-cultured RGD films using a two-color fluorescence viability assay. Adhered cells were found on all matrices after 24 h, with almost no dead cells. Additionally, at an early time point of 3 h, the cells on the RGD membrane showed many distinct focal adhesion points and well-organized actin stress fibers, which were similar to those on fibronectin, indicating an excellent cell attachment activity [26]. However, the cell types and their sensitivity to growth conditions should certainly be considered with respect to the evaluation of RGD-based materials for adhesion [27].

To explore the interaction between cells and morphology/topology of various forms of spider silk-based materials, Aldo et al. characterized the films, hydrogels, and electrospun nonwoven meshes made of the recombinant spider silk protein eADF4(C16) on the adhesion and proliferation of fibroblasts. The films and hydrogels had high hydration and a low contact angle. They produced a thin layer of water on their surface, which inhibits proteins' adhesion, reducing cell attachment. In comparison, the nonwoven meshes displayed a defined surface topology of high surface area, which is conducive to the filopodia protrusion and the posterior spreading around the fibers. This phenomenon usually occurs in the absence of specific binding sequences/ligands to enhance cell adhesion. With the increase in fiber diameter, the distance between fibers also increases, making the filopodia protrude more efficiently [28].

### 2.3. Mechanical Properties of Spider Silk: From Sequence to Structure

**Figure 3:**
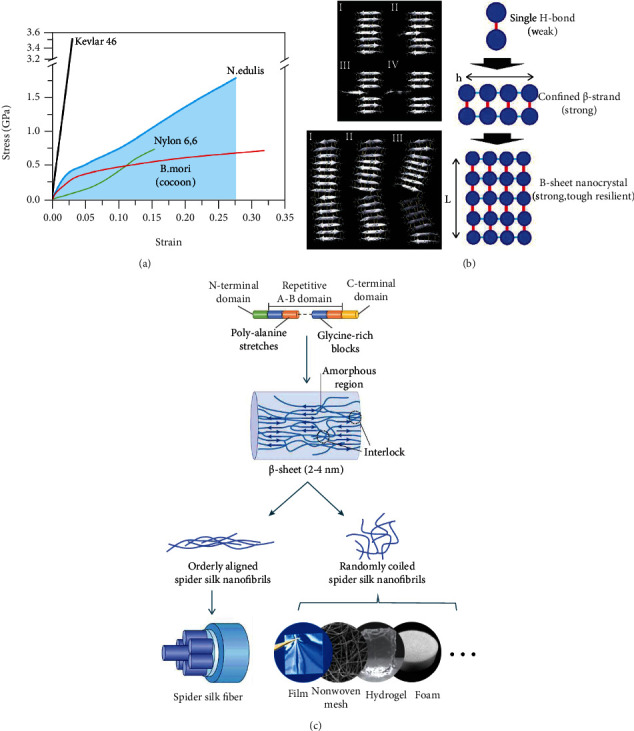
(a) The strain-stress curve of different fiber materials including Kevlar 46, dragline fiber of *N. edulis*, Nylon 6,6, and cocoon silk fiber from *B. mori*; the toughness of *N. edulis* dragline fiber is illustrated by a colored area. Reproduced from C. Fu et al., 2009, Copyright © 2009, Royal Society of Chemistry. (b) Hierarchical organization of spider silk nanocrystals and deformation profiles of *β*-sheet crystals at different sizes. Reproduced from S. Keten et al., 2009, Copyright © 2021, Nature Publishing Group. (c) Hierarchical structure of spider silk fiber and nonfiber spidroin materials. Based on the nanofibril network topology and internanofibril interaction strength, silk fibers and nonfiber silk materials are of different fibril arrangements: among silk fibers, silk nanofibrils are bundled along the fibrous axis, while for nonfiber silk materials, silk nanofibrils are interconnected in a nearly random manner. Reproduced from S. Ling et al., 2018, Copyright © 2021, Macmillan Publishers Limited and W. Qiu et al., 2019, Copyright © 2021, Wiley-VCH Verlag GmbH & Co. KGaA, Weinheim.

Natural spider silk, especially the major ampullate silk, has impressive comprehensive mechanical properties, including high tensile strength (0.9~1.4 GPa) and excellent extensibility (25%~40%), which endow spider silks with excellent toughness (colored area under the curve in Figure 3(a)), 160~250 MJ cm^−3^) which is even higher than that of steel and Kevlar fibers [7, 29, 30]. In addition to the excellent comprehensive mechanical properties, the fine hierarchical structure of spider silk has also been widely researched. The well-known dragline silk is mainly composed of two fibrous proteins MaSp1 and MaSp2, which are secreted from the major ampullate silk gland. According to the amino acid sequence, the dragline spidroin molecular chain is composed of three blocks, including two nonrepetitive domains N-/C-terminals and repetitive regions made of poly-alanine stretches (A_*n*_, *n* = 4-10 for MaSp1 and *n* = 3-9 for MaSp2) and glycine-rich blocks (GGX (*X* = *A*, *Q*, or *Y*) and GX (*X* = *Q*, *A*, or *R*) for MaSp1 and GPX (*X* = *G* or S), QQ, GGX (*X* is usually *A*), and GSG for MaSp2) [31–33]. During the natural spinning process, N-/C-terminals perform a response to pH gradient, dehydration, ion exchange, and shear stress, thereby inducing the assembly of aligned protein molecular chains and the elongation of silk fibers [34].

On the molecular scale, poly-alanine (A_*n*_) stretching compounds are pressed together and tightly connected through H-bonds to form the hard *β*-sheet nanocrystals with high modulus. Poly-alanine stretching compounds commonly include 4-12 Ala with 1-3 nm in length (*h* = 1~3 nm in Figure 3(a)), and the nanocrystal size is precisely controlled at a critical length about 2-4 nm (Figure 3(c)). When nanocrystals of this size are tensioned, the molecular chains begin to slip stickily due to the hydrogen bonds breaking down and regeneration in this process. These phenomena not only dramatically increase the total dissipated energy of fibril deformation but also prevent the internal defects of nanocrystals from being exposed to the surrounding water and finally result in high strength, stiffness, and toughness. For nanocrystals with length larger than critical length, nanocrystals tend to be in bending mode rather than the favorable slipping mode during tension. The crack caused by unbalanced stress will be attacked by surrounding water, which leads to large-scale breaking down of hydrogen bonds followed by fiber failure [35, 36]. As shown in Figure 3(c), glycine-rich (GGX) blocks of each protein molecular chain have *α*-helix and random coil structure acting as the amorphous region within spider silk nanofibrils and established elastic connections between *β*-sheet nanocrystals. When the fiber is stretched, the amorphous region will deform due to its low modulus, and the interlocking between molecular chains effectively limits the crack propagation. Therefore, the amorphous regions composed of *α*-helix and random coil are generally regarded as the main contribution to the elasticity and ductility of spider silks [37].

The complex *β*-sheet nanocrystal and amorphous region network form spider silk nanofibrils, and the crosslinking ways of these nanofibrils will ultimately determine the product formation. For natural and artificial spider silks, fibers are spun from silk glands or artificial spinning facilities. Under the traction of the spinning process, bundles of nanofibrils are orderly aligned along the long axis of the fiber. A large number of interlocking structures between nanofibrils further strengthen the toughness of the nanofibril network and the whole spider silk fiber. Furthermore, the self-healing outer layers of natural spider silks made of lipid and glycoprotein act as shields that protect core fibers from the invasion of water, protease, and environmental microorganisms [38, 39]. As for the 2D and 3D formation of spider silk-based biomaterials, various preparation methods can be utilized via controlling the distribution and self-assembly behavior of randomly crimped spider silk nanofibers, thereby adjusting the mechanical properties to satisfy the requirement of tissue regeneration in different locations.

## 3. Spidroin-Based Biomaterials in Different Dimensions

### 3.1. One-Dimensional (1D) Spidroin-Based Biomaterials (Fiber)

The spider silk fiber is a unique biomaterial with excellent mechanical properties. Compared with silk from the silkworm, spider silk shows better biocompatibility due to the lack of sericin coating, which is a kind of high immunogenic protein that may cause serious hypertensives [40–43]. In tissue engineering, natural spider silk is usually collected from living spiders. More researchers also use recombinant spidroins to prepare recombinant spider silks through artificial spinning methods. Additionally, spider silk fibers are usually orientated in parallel as a scaffold to guide the regeneration of fibrous tissues and cells along the fiber direction [44, 45]. According to the research from Fredriksson et al., the implanted recombinant 4RepCT spider silk fiber illustrated better ability that promotes ingrowth of fibroblasts and formation of new capillaries in rats compared to the commercial medical implant Mersilk™ fiber, which shows the great potential for spider silk fiber as scaffold material in tissue engineering [46].

The strain-stress curve of spider silk illustrates a similar variation tendency like that of the tendon, and the curve has both low-strain modulus and high-strain modulus stages. Interestingly, spider silk also shows the shape memory effect which allows it to recover the initial form after deformation, such as tendon [47, 48]. Hennecke et al. reported their study of using bundles braided from natural spider silk single fiber (from female adult *Nephila* spiders ranging in age from 6 to 15 months) as sutures for tendon surgery and characterized the mechanical properties of these sutures produced using different braiding methods. Compared with conventional sutures, spider silk sutures demonstrated an excellent fatigue response and maintained high tensile strength compared to conventional ones even after 1000 fatigue cycles without any reduction [49].

**Figure 4:**
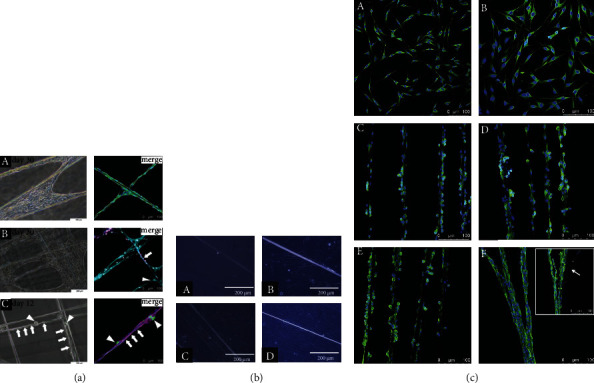
Adhesion of different cells on spider silk fibers. (a) Representative phase contrast versus confocal and immunostaining images of (A) rSCs, (B) rFBs, and (C) rDRG neurons cultured on spider silk. The merged channels of rSCs were S100 (green), VIME (grey), and DAPI (blue); the merged channels of rFBs were NGFR (magenta), THY1 (cyan), and DAPI (grey); and the merged channels of rDRG neurons were TUJ1 (beta-3-tubulin) (green), NGFR (magenta), and DAPI (blue). Arrows (left) indicate elongated rDRGs along the silk fibers, and arrows (right) indicate rFBs and rDRG neuron bodies. Reproduced from F. Millesi et al., 2021, under the Creative Commons Attribution License. (b) Images showed the proliferation and alignment of DPSCs on the spider silk over time (10x magnification). Reproduced from K. Hafner et al., 2017, Copyright © 2021, Elsevier B.V. All rights reserved. (c) Immunostaining images of rSCs cultured on (A and B) PLL/laminin-coated dishes and (C) dragline, (D) cocoon, (E) connecting, and (F) attaching silk showing S100 (green) and DAPI (blue) staining. The white arrow points towards the single silk strands that were barely perceivable. Reproduced from A. Naghilou et al., 2020, under the Creative Commons Attribution License.

Due to the outstanding tensile strength, good elasticity and long-term biodegradability of natural spider silk, especially the favorable quick adhesion of cultivated Schwann cells on silks, spider silk has become an ideal biomaterial for the construction of nerve scaffolds [50]. Radtke et al. reported their research about sheep tibial nerve defect reparation using nerve constructs composed of decellularized vein grafts and spider silk fibers. The experiment results exhibited that spider silk fibers can promote nerve tissue regeneration, axon regeneration, remyelination, and potential Schwann cells recruitment ability [51]. Roloff et al. firstly described in detail the adhesion and growth condition of human (NT2) model neurons on crossed spider silk fiber scaffolds *in vitro*. Human neurons cultured on spider silk arrays were grown along fiber direction due to the increased migration and adhesion. The newly extending neurites bridged gaps between single fibers and were elongated on neighbor fibers. Within three to four weeks, the neuron cluster and ganglion-like cell structures were observed, suggesting that the spider silk could be an excellent candidate biomaterial for enhancing neural repair in the injured central nervous system because of the neuron affinity and nonimmunogenicity of the spider silks [52]. The research from Millesi et al. demonstrated a similar result that native spider silk fibers possess excellent adhesive properties and promote cell alignment, proliferation, and migration, as shown in Figure 4(a). Native spider silk fibers provide framework SCs to form Bungner bands for regrowing axons by supporting the repair of the inherent properties of SCS, replacing the basal laminar tube as a terrain guiding structure [53]. To further explore the potential of spider silk in tissue regeneration of the nervous system, Hafner et al. evaluated the response of human dental pulp stem cells (DPSCs) to spider dragline silk fibers, and the result is illustrated in Figure 4(b). The outcomes implied that the spider silk fiber substrate may induce stem cells to differentiate into more extended cell morphology lineages for subsequent neural differentiation applications [44].

The study from Naghilou et al. partially explained the induction mechanism of spider silk fiber on cells. They collected natural dragline silk and cocoon silk from *N. edulis* and the connecting and attaching silk from *A. avicularia*, investigated the material characteristics of each kind of silk samples, and correlated their ability to act as a guiding structure for rat Schwann cells (rSCs) during *in vitro* experiments. Compared to other three kinds of spider silk fibers, the *β*-sheet content of attaching silks decreased significantly, which led to severe adhesion of fibers in the medium and the production of cell aggregates. Meanwhile, the successful guiding behaviors of rSCs on dragline silk, cocoon silk, and connecting silk are observed, as shown in Figure 4(c). The experiment result exhibited the necessity of higher *β*-sheet content in spider silk fiber for fabricating nerve guidance conduits by guaranteeing the fiber to be straight and stiff in the medium [54].

Kornfeld et al. repeated the sheep tibial nerve defect reparation experiment using the similar spider silk-based implant, quantified the axonal regeneration rate, and determined the *in vivo* degradation mechanism and rate of spider silk fibers. Surprisingly, the axonal regeneration rate occurred on a spidroin-based implant with a directional velocity of 1.309 mm/d, close to that of autologous nerve transplant (1.57 mm/d, the ideal axonal regeneration rate). The implanted spider silks could be fully degraded through the photoimmune response mainly mediated by Langerhans giant cells within 3 months, so that the constructs could be replaced by an autologous matrix [55].

### 3.2. Two-Dimensional (2D) Spidroin-Based Biomaterials

#### 3.2.1. Nonwoven Mesh

Electrospinning is widely used in tissue engineering and usually has good compatibility with biological tissues. Unlike the weaving method, electrospinning can produce ultrathin nonwoven textile with a large specific surface [56, 57] and even mimic the histological structures through electrospinning process design [58]. Limited by the yield of natural spidroin, the recombinant spidroin produced by genetically engineered bacteria (e.g., *Escherichia coli*) or yeasts (e.g., *Pichia pastoris*) is adopted as raw material [59]. In order to inhibit the self-assembly of spidroin molecular chains, the strongly polar solvent hexafluoroisopropanol (HFIP) and formic acid are generally selected for distributing purified spidroins [60]. However, a more mild and biocompatible Tris/HCl buffer plus PEO400 is suggested as a solvent for biomedical use nonwoven mesh to avoid the tissue irritation brought from polar solvent [61]. After spinning, posttreatment is also required, such as methanol or ethanol steam bath, since they can transform *α*-helix structures into *β*-sheet structures to endow mesh with better water resistance and water stability [60, 62].

Leal-Egaña et al. examined nonwoven mesh, film, and hydrogel scaffolds made of eADF4(C16) and evaluated the cell adhesion and proliferation on these substrates. Among the three candidate materials, the nonwoven mesh showed the highest potential on fibroblast adhesion and proliferation, which was attributed to the higher availability of cell attachment surface in the daedal surface structure of nonwovens, allowing faster production of filaments around fibers. Furthermore, their experiment also described the proper fiber diameter ranges, i.e., ideal cell adhesion of 500~1500 nm and good proliferation of 1000~2000 nm [28]. This research provides valuable information for the surface design of nonwoven mesh. The research about using pNSR16/PVA nonwoven mesh as a wound dressing to accelerate wound healing was reported by Zhao et al. [63]. During the healing period, the nonwoven mesh could effectively promote cell adhesion, proliferation, and migration, and faster formation of granular groups and wound contraction were also observed in the experimental group.

In addition, the recombinant spidroin-based nonwoven mesh also has broad application prospects in the field of nerve repair. In the study of Pawar et al., aligned collagen fibers were packaged with eADF(C16) nonwoven mesh to prepare a nerve catheter, and the spidroin shell was also chemically modified to enhance its cellular accessibility. In this research, the aligned fibers caused no cell toxicity during the 3-week incubation period. The surface structure of spidroin mesh provided ideal attaching sites for cells required for nerve regeneration. This porous shell also maintained the transportation of nutrition, gases, and waste metabolites around nerve defects [64].

#### 3.2.2. Film

Spidroin films can be manufactured through the conventional film-forming methods, e.g., casting and spin coating, and the choice of solvent will significantly influence secondary structure change in the film; for instance, the films made from formic acid or aqueous solution demonstrate higher *β*-sheet concentration, while using HFIP as solvent will lead to high *α*-helix concentration in the film after solvent evaporation and posttreatments are required [60, 65, 66]. Generally, the water annealing (121°C hot steam treatment), salt solutions (e.g., 1 M K_2_PO_4_ solution), and primary alcohol solution or steam are used to transform part of *α*-helix into *β*-sheet to enhance the water stability and mechanical properties of films [18, 67, 68].

**Figure 5:**
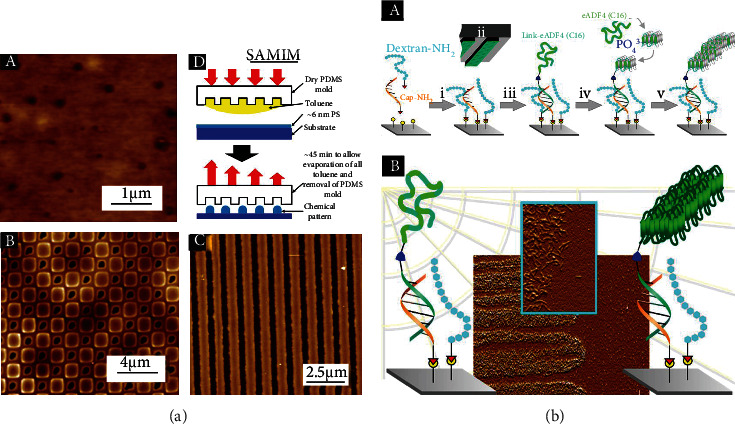
Using different techniques for patterning materials of recombinant spider silk proteins. (a) AFM images showing the morphology of C16 films from HFIP: (A) surface morphology with a *Z* scales of 10 nm, (B) square pattern with a *Z* scale of 60 nm, (C) line pattern using the SAMIM technique with a *Z* scale of 60 nm, and (D) representation of the SAMIM technique. Reproduced from S. L. Young et al., 2012, Copyright © 2012, American Chemical Society. (b) (A) Schematic representation of soft lithography-based patterning for localized fibril self-assembly. The silicon surface was activated by the epoxy group, enabling the simultaneous introduction of the capture 5′-amino oligonucleotides (cap-NH_2_) and passivation agents (i). A microcontact printing technique (ii) was used to pattern DNA-spider silk conjugates (link-eADF4(C16)) (iii). The surfaces were treated with phosphate ions and monomeric spider silk protein eADF4(C16) to nucleate (iv) which led to the growth of surface bound *β*-sheet rich fibrils on the spotted conjugates. (B) Schematic diagram of surface microstructure formed by self-assembly of spidroin molecular chains. Reproduced from A. Molina et al., 2019, Copyright © 2018, American Chemical Society.

Various physical-chemical and genetic modifications can be applied to the film's surface structure to achieve better cell adhesion and cell fate control or to import specific properties. Micropatterns could improve cell adhesion and adjust cell orientation on the film surface [28, 69]. Meanwhile, micropatterns on the film surface can also prevent microbial infestation as antibacterial layers [70]. Commonly, microscale patterns are created through capillary transfer lithography (CTL) and solvent-assisted microcontact molding (SAMIM) using a PDMS mold as shown in Figure 5(a) [70, 71], and Figure 5(b) illustrates the research from Molina et al., where they reported a technology utilizing the directing ability of complementary DNA strands and spidroin self-assembly induced by phosphate ions to form nanoscale patterns on the inorganic surface [72].

Besides surface micropatterns, different kinds of cell bonding peptides were also researched to promote the cell adhesion capability of the spidroin molecular chain. The integrin-binding motif RGD from fibronectin was firstly investigated and connected to the spidroin sequence through genetic editing. The RGD-modified film surface demonstrated enhanced cell adhesion, which may be due to the interaction of nonspecific cells in protein molecules with charged residues [26, 73, 74]. Furthermore, different cells also illustrated various responses on different motif-modified films. For example, Schwann cells have been shown to adhere to matrices with IKVAV motifs, with dispersed morphology and improved viability [26, 75]. Silk materials can also be functionalized with oligosaccharides, acrylates, and other synthetic polymers to improve cell adhesion [76]. The alkyne-capped-PMAGal derivatives were linked with the azidopropylamine-modified eADF4(C16) film, showing up to 80% cell attachment compared with 69% of the unmodified membrane [77].

### 3.3. Three-Dimensional (3D) Spidroin-Based Biomaterials

#### 3.3.1. Hydrogel

Hydrogels are three-dimensional (3D) crosslinked polymer networks with high water content (above 95% *w*/*w*) and high adsorption capacity [78, 79]. Various natural and synthetic-derived polymers, such as sodium alginate, collagen, hyaluronic acid, chitosan, silk, gelatin, polyvinyl alcohol (PVA), and polyacrylic acid (PAA), have been processed into hydrogels via physical or chemical crosslinking for a wild range of biomedical applications [45, 80]. Due to the excellent mechanical properties and similarity to ECM, hydrogels based on spider silk proteins have been widely explored and utilized, especially in the fields of tissue engineering, drug delivery, and biomanufacturing.

The hydrogel structure can be generated from spider silk protein solutions through a sol-gel transformation. In the process of gelation, spider silk proteins are driven by hydrophobic interactions and hydrogen bonds to self-assemble into *β*-sheets [81–83]. Generally, adjusting physical crosslinking conditions, such as the concentration of the spidroin solution and additives, pH value, ionic composition of the solution, or shear force, can control the gelation time and enhance the stability of the hydrogel [84]. Schacht and Scheibel explored a duplicable approach for hydrogel formation of engineered and recombinantly produced the spider silk protein eADF4(C16), in which the low-concentration protein was denatured in 6 M GdmSCN and then dialyzed against 10 mM Tris/HCl (pH 7.5). With increasing protein concentration, the formation of *β*-sheet-rich nanofibrils of eADF4(C16) is accompanied by gelation [85].

The use of chemical crosslinkers is also an effective method to stabilize the three-dimensional structure of hydrogels. To prevent hydrogel from being damaged by agitation or shearing, Rammensee et al. treated the surface of C16 hydrogels with ammonium peroxodisulfate (APS) and tris(2,2′-bipyridyl)dichlororuthenium (II) (Rubpy) to connect the tyrosine side chains of the spidroins. The shear modulus of the noncrosslinked C16 silk hydrogel and crosslinked hydrogel was about 38 Pa and 820 Pa, respectively [86]. Neubauer et al. demonstrated the recombinant spider silk protein eADF4(C16) assembly into fibrils in the presence of DMSO/DMF in water-soluble organic cosolvents. The tris-hydrogels and gels in DMSO blend exhibited the highest *β*-sheet content (39 ± 1%), whereas the gels in the presence of water and DMF blends showed far fewer *β*-sheets of 24 ± 5% and 22 ± 4%, respectively. In addition, the above-mentioned recombinant spider silk hydrogel exhibited excellent shear-thinning characteristics with respect to the protection of encapsulated cells or drugs from corrosive shear stress. The authors further used the 3D printing method to fabricate multilayer scaffolds with high shape stability, which could be applied to a 3D printing drug storehouse [87]. The addition of nanoparticles has recently been proven to be an important factor in enhancing hydrogels' adhesion and mechanical properties [88]. Liu's group reported on artificial spider silks produced by the water evaporation-induced self-assembly of hydrogel fiber consisting of polyacrylic acid and silica nanoparticles. The mechanical properties of the ultimate fiber were comparable to those of spider silk, presenting a tensile property of 44.3%, a tensile strength of 895 MPa, a toughness of 370 MJ m^−3^, and a damping capacity of 95% [89].

#### 3.3.2. Porous Sponge

Matrix stiffness is increasingly appreciated as a critical mediator of cell behavior to regulate cell signaling, with effects on growth, survival, and motility [90, 91]. Hydrogel showed poor stability in cell and tissue culture, whereas the 3D scaffolds with a particular content of stiffness can promote cell adhesion and proliferation, generate a new extracellular matrix, promote tissue growth, and promote the transport of nutrients and metabolites. Although a number of methods have been used to prepare silk-based scaffolds [17, 92–95], the salt leaching method is widely used due to its effectiveness, efficiency, and ease of the process. NaCl crystals allowed the production of *β*-sheet-rich scaffolds with a high porosity without the need of additives or crosslinkers for mechanical stabilization, as well as leaving a gap in practicability for some soft tissues. Therefore, choosing a porous and mechanically stable silk salt-leached matrix to match the stiffness of soft tissue will provide additional options for developing spider silk biomaterials in soft tissue regeneration.

In Agapov's study, the three-dimensional scaffold based on recombinant protein 1 was produced by leaching. After 24 h of cultivation, the cells were mainly distributed on the surface of the scaffold, and only a few cells were detected at depths over 200 *μ*m from the scaffold surface. The cell density averaged for all layers was 355 ± 64 cells per 1 mm of the scaffold. After 4 days of cultivation, fibroblast nuclei were detected in the deep pore wall of the scaffold, indicating that the scaffold had good compatibility and could provide efficient cell adhesion and proliferation over a long time period [96]. Moisenovich et al. have previously generated porous scaffolds from a genetically engineered protein, an analog of *N*. *clavipes* spidroin 1 known as rS1/9. The salt leaching technique was used to make the rS1/9 scaffolds of interconnected porous structures with spontaneously formed micropores. The 3T3 fibroblasts proliferated in the rS1/9 scaffold and distributed uniformly between the surface and deeper layers (about 27% and 33%, respectively), whereas the initial distribution was about 58% and 11%, respectively [97]. In the 8-week subcutaneous transplantation experiment, compared with the control group, the nerve fibers on rS1/9 scaffolds showed deeper penetration in the process of vascularization and regeneration of connective tissue in BALB/c mice [98].

Surface modification of various biomaterials is a promising method to endow the biofunctionality at the material-tissue interface to modulate biological responses [99, 100]. Schacht et al. fabricated highly porous foams made of the recombinant spider silk protein eADF4(C16) containing an RGD-binding motif to prepare highly porous foam through the salt leaching method, thereby obtaining mechanically robust scaffolds. The results showed an improved adhesion and proliferation of fibroblast on eADF4(C16)-RGD foam, indicating the potential applications in soft tissue engineering and tissue repair [101]. These studies confirmed that the combination of salt leaching and spidroins pretreatment was a practical approach to tune the scaffolds to produce a mechanical system closer to softer natural tissues.

## 4. Spidroin-Based Biomaterials for Regenerative Therapies

The existing knowledge of spidroin-based biomaterials in terms of structure and function provides a theoretical basis for the design and synthesis of new spider silk protein biomaterials. In addition, genetic engineering techniques can also be used to add gene sequences of new peptides or domains to the coding sequence of spider silk proteins to endow new functions. In this section, we discussed the potential of genetic engineering of spider silk protein in the preparation and drug delivery of lung tissue engineering and also discussed the application status of combining stem cells and spider protein-based biomaterials in the field of tissue engineering.

### 4.1. Lung Tissue Engineering

**Figure 6:**
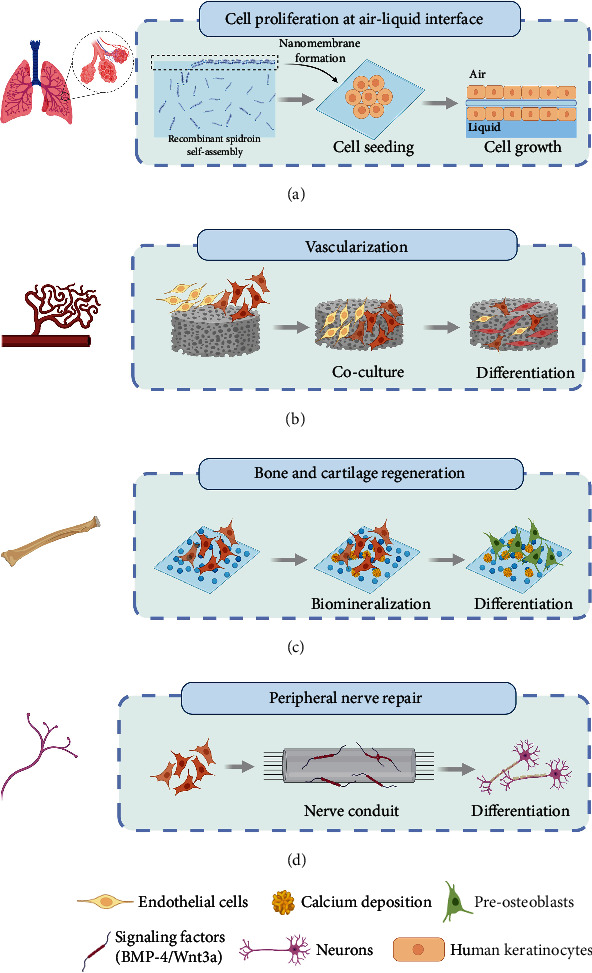
Schematic illustration of spidroin-based biomaterials for various tissue engineering applications. (a) For lung tissue engineering, FN-4RepCT silk protein forms a layer of nanofibrils via self-assembling at the liquid-air interface. The membrane enables the rapid formation of a confluent cell layer of human keratinocytes on either side. (b) For vascularization, the endothelial cells (ECs) and mesenchymal stem cells (MSCs) were cocultured on the recombinant spider silk (4RepCT) foams, and it was found that MSCs grew millimeter long branched sprouts in the foam within two weeks. (c) For bone and cartilage regeneration, the bone marrow mesenchymal stem cells (hMSCs) cultured on recombinant protein films can promote osteogenic differentiation by enhancing biomineralization. (d) For peripheral nerve repair, the neural stem cells (NSCs) cultured on the 4RepCT matrices differentiated efficiently into neurons after processing the recombinant protein including the extracellular signaling factor BMP4 or a combination of BMP4 and the signaling factor Wnt3a.

An effective strategy for the development of the tissue-engineered lung is to simulate the architecture of capillary conduits in the lung tissue, which are covered with monolayer epithelial cells and have a large surface area conducive to gas diffusion between the external environment and blood [102]. The spider silk membrane (thickness > 3 *μ*m) has been extensively investigated for the replacements of the retinal pigment epithelium, bone grafts, and corneal implants due to its biocompatibility, degradability, and molecular permeability, but this microscaled structure is not suitable for the replacement of ultrathin alveoli tissue. Bearing in mind such limitations, Gustafsson et al.'s group fabricated ultrathin nanofilms (~250 nm) based on the self-assembly of recombinant spider silk proteins at the liquid-air interface of a standing aqueous solution (as shown in Figure 6(a)). The nanomembrane displayed a high elasticity and toughness, contributed to the permeability of human plasma proteins, and promoted cells to form a confluent monolayer within three days [103]. In addition, the thickness of nanofilms could be tightly controlled by adjusting silk concentration, and those well-designed structures show encouraging prospects in terms of the cocultured system with the different types of cells, which opens the door to develop an *in vitro* lung tissue model based on the coculturing cells upon the ultrathin membrane [104].

Pulmonary surfactant (PS) is a surface-active lipoprotein complex with hydrophilic and hydrophobic properties, which reduces the expansion force by adsorbing on the liquid and air interface of the alveoli, thereby avoiding the alveoli collapse. Nowadays, the lack of PS has been proven to be the leading cause of neonatal and pediatric respiratory diseases, so the research and development of PS artificial preparation have become a research hotspot. However, PS is prone to aggregate due to the unstable secondary structure [105], which may severely impede the production of recombinant PS proteins. In the process of producing high-viscosity spider silk proteins, spiders will separate protein regions with aggregation tendency into micellar structures, and the shell of micellar structure is the N-terminal domain (NT) of protein. Inspired by this idea, Kronqvist et al.'s group designed a mutant spider silk protein domain, NT∗, exhibiting markedly increased solubility, stability, and refolding capacity. They further developed the recombinant PS with an NT∗ fusion tag, which showed low cost, high yield, and few purification steps. By comparing the synthetic PS with the analog biological products on the market, it is found that it can effectively reduce the lung surface tension of the animal model of infant respiratory distress syndrome, which provides a promising prospect for the artificial production of low-cost PS for the treatment of various lung diseases [106].

Compared with tissue engineering scaffolds for other organs, besides the conventional requirements for scaffolds, lung tissue engineering puts forward higher needs, such as sufficient mechanical strength and elasticity to provide tensile stress similar to the natural lung, thereby offering the necessary distensibility of the lung when breathing. Meanwhile, an appropriate microcirculation structure is also required to achieve gas and nutrition exchange. Surprisingly, the research of Gustafsson et al.'s and Kronqvist et al.'s groups has achieved partial functions that scaffolds for lung tissue engineering need, including recombinant pulmonary surfactant and cell proliferation at the air-liquid interface, which indicates the great possibility of using a spidroin-based scaffold for lung tissue engineering.

### 4.2. Vascularization

The investigation of tissue-engineered vascular constructs (TEVs) is being vigorously pursued by converging multidisciplinary approaches in recent years. The goal of producing engineered blood vessels is to construct a living neovessel with similar architecture and biological functions to the native blood vessel. However, the artificial blood vessels in the early phase generally exhibited instability due to the lack of vascular resistance and poor mechanical strength of the vessel walls. Spider silks have been shown in various studies due to their improved mechanical properties, inherent biocompatibility and biodegradability, relative ease of processing [49, 107], and weaker immunological reactions compared to other materials (e.g., PLA and collagen) [108]. These features recommend spider silk-based scaffolds as suitable alternatives for the development of vascular tissue engineering. Although a variety of cell types have been utilized to fabricate TEVs, approaches to using stem cells can overcome the limitations of using differentiated vascular phenotypes and reduce the preparation time of TEVs. Johansson et al. developed 3D foams based on a recombinant spider silk protein (4RepCT; consisting of the C-terminal domain and four tandem repeats of the major ampullate spidroin-1). The pluripotent and multipotent stem cells were seeded on the 3D culture to investigate their applicability for efficient differentiation. The outcomes demonstrated that the human embryonic stem cells (hESC) were aggregated and integrated within the 3D structure of silk foam after culturing 48 h. Endodermal differentiation was further examined by the mRNA expression experiment, confirming robust upregulation of the endodermal progenitor marker SOX17 and downregulation of pluripotency (NANOG). Figure 6(b) shows a further examination of organizational capacity for generating microvessels; the endothelial cells (ECs) and mesenchymal stem cells (MSCs) were cocultured, and it was found that MSCs grew millimeter long branched sprouts in the silk within two weeks [109].

### 4.3. Bone and Cartilage Regeneration

The mechanical strength, resilience, and flexibility of spider silk are superior to those of other natural polymers and most synthetic materials, exhibiting even high-performance fibers such as Kevlar, holding great promise for the development of a bone-related functional scaffold [110]. Many studies have focused on enhancing bone regeneration of spider silk-based composite materials by improving the mineralization of the bone matrix. Dinjaski et al. fused the hydroxyapatite-binding domain VTKHLNQISQSY (VTK) separately to the N-, C-, or both terminals of the spider silk to investigate the effect of VTK position on the properties of biomaterials and biomineralization. The hMSCs were cultured on the surface of the silk-VTK fusion protein films to verify the effect of the recombinant protein membrane on promoting osteogenesis *in vitro*. As shown in Figure 6(c), the elongated morphology of hMSCs was observed on all test surfaces after 14 days, indicating that hMSCs grew healthily and an absence of cytotoxicity to the recombinant silk-VTK proteins. The outcomes also showed that the recombinant fusion harboring VTK domains on both the N- and C-termini produced the highest amount of bone sialoprotein deposition and calcium deposition, indicating the potential to induce hMSC differentiation [111]. Hardy et al. fabricated films consisting of poly(butylene terephthalate) (PBT) or poly(butylene terephthalate-co-poly(alkylene glycol) terephthalate) (PBTAT) and an engineered spider silk protein (eADF4(C16)). The calcium phosphate mineralized films possessed multiple carboxylic acid moieties capable of binding calcium ions, which can facilitate biomineralization with calcium carbonate or calcium phosphate. Qualitative analysis of alkaline phosphatase (ALP) activity showed that hMSCs on the membrane could differentiate into osteoblasts [112]. The authors further explored the effect of photochemical modification on spider silk protein-based films. The eADF4(C16) films were firstly immersed in pure monomers (e.g., allylamine, acrylic acid, and methacrylic acid), exposed to ultraviolet light for 48 h, and then washed and dried in a vacuum. This approach led to the generation of free radicals on the silk protein backbone and produced a functional polymer coating after grafting the synthetic polymers, thereby further enhancing the mineralization of calcium carbonate [113].

### 4.4. Peripheral Nerve Repair

In the clinical management of nerve gap repair, the treatment effect of injured patients is not as good as expected. Effective repair also becomes progressively more difficult with the increase in the nerve gap. To overcome these limitations, nerve conduit strategies based on spider silk scaffolds were proposed to meet the clinical demands and requirements of large-gap nerve repair. Lewicka et al. proved that the recombinant spider silk protein 4RepCT offered a suitable substrate for the culture of rodent cortical neural stem cells (NSCs). These cells can proliferate, survive, and efficiently differentiate into a variety of cell types on the 4RepCT-based membrane structure without any additional coating or animal-derived components [114]. They further processed recombinant proteins with the extracellular signaling factor BMP-4 or a combination of the signaling factor Wnt3a and BMP-4. As shown in Figure 6(d), it was found that cultured cortical NSCs in 4RepCT foam-like structures could be effectively and efficiently differentiated into neurons responsive to glutamate receptor agonists (such as AMPA) [115]. Astrand et al. created a centimeter-sized foam as an ideal stem cell niche by using recombinant spider silk protein modifying fibronectin- (FN-silk-) binding motif and a human recombinant laminin 521 (LN-521). In this 3D structure, human pluripotent stem cells (hPSC) developed into 3D cell constructs supported by a microfibrillar network. After the initial expansion, neurons will be induced to differentiate into a continuous layer of neuroectodermal tissue and further differentiate into neuronal subtypes [116].

Another ideal source of stem cells proposed for neural regeneration is DPSCs because of their close relationship with the embryonic origin of neurons and easily available properties. Hafner et al. explored the effects of dragline silks on cellular attachments and proliferation of DPSCs. The outcomes demonstrated that the spider silk fibers have good biocompatibility and are free of an immunogenic sericin coat [117]. The *in vitro* cell experiment results showed that DPSCs had a larger tendency of arrangement and extension on the spider silk matrix, while fibroblasts and osteoblasts had a smaller tendency of arrangement and extension, suggesting that these fibers may also be recognized as an effective matrix for DPSCs to differentiate along more elongated cell morphology under appropriate growth environment [44].

## 5. Conclusion and Perspective

Good biocompatibility, nonimmunogenicity, biodegradability, and comprehensive mechanical properties endow spidroin material with great potential in tissue engineering. At present, a small amount of spidroin can be obtained directly from nature. The use of genetically modified bacteria or fungi is still the primary way to produce recombinant spidroin on a large scale. Recombinant spidroin can be processed into materials of different dimensions to meet the requirements of different tissue morphology, structures, and mechanical properties. This review briefly introduced the characteristics of spidroin materials, the preparation methods of different dimensional scaffolds, and their applications in lung tissue engineering, vascular tissue engineering, bone tissue engineering, and nerve repair tissue engineering. Although there are few *in vivo* studies of spidroin materials in the lung tissue engineering area, we still make a brief overview and summary. For the spidroin-based scaffold used for different tissue engineering, there are still a lot of functions and characteristics to be realized, such as the complex multilevel structure and stability and selective permeability under certain mechanical properties. We still firmly believe that these goals can be achieved by applying new manufacturing methods and further understanding the relationship between the structure, component, and properties of spidroin-based biomaterials.

The ECM of each organ possesses a very complex and unique structure, components, and unique cell regulatory mechanism. Therefore, in order to fully simulate the structure and function of the engine control module, it is necessary to design a multidimensional framework structure to realize the multilevel regulation of cell growth, differentiation, and migration. The framework material can respond to multiple fields (such as force, heat, light, electricity, and magnetism) to enhance the interaction between scaffold and tissue cells. In the future, a variety of processing methods can be conjunctively used to realize the precision manufacturing of complex structures, which is conducive to the preparation of complex structures with performance gradients and anisotropy [118, 119]. For example, 4D printing technology can make the scaffold respond to stimulation and provide more ways for cell fate regulation, which involves a full understanding and fine control of various components of spider silk protein.

In addition, the ultrahigh production cost of recombinant spidroin may become the major obstacle to the industrial production and large-scale application of the recombinant spidroin-based fiber or scaffold. As AMSilk [120] (a German company specializing in high-performance spidroin-based biopolymer) claimed in 2011, the cost of per-kilogram of recombinant spidroin is around €100,000 (~$137,500) if there is no complete reinvention on production processes, and it is also close to the estimation from Kraig Biocraft Lab [121] in 2018 that the high yield of 20 grams of protein per liter of fermentation broth is $35,000~$37,500. Meanwhile, the environmental and health impacts and costs 5of solvents in the manufacturing process cannot be ignored. The corrosive, irritant, and environmentally harmful solvents (e.g., HFIP [28, 64], GdmSCN [74, 122], and formic acid [63]) are frequently adopted to dissolve recombinant spidroins for subsequent procedures due to their excellent ability to anti-self-assembly of spidroins, which definitely increases the operational risk and environmental issues. Fortunately, there is a new trend that uses gentle and environmentally friendly aqueous solvent when preparing a spidroin-based film or scaffold used for tissue engineering [57], such as Tris-HCl, SDS, and PEO400. Correspondingly, increasing the protein concentration within these solvents becomes a new challenge. Based on the above reasons, preparing polymers to simulate the various characteristic components and hierarchical structure of natural spider silk through chemical synthesis methods will also become a research hotspot, which is expected to reduce the production cost of preparing artificial spider silk significantly [123, 124]. However, the biocompatibility, degradation performance, immunogenicity, and processability of artificial spider silk protein prepared by chemical methods still need to be further studied.

## Data Availability

The figures and data supporting this review are adopted from previously reported studies and datasets, these studies have been cited, and permissions have been obtained.
